# Cold-Active Enzymes and Their Potential Industrial Applications—A Review

**DOI:** 10.3390/molecules27185885

**Published:** 2022-09-10

**Authors:** Burhan Hamid, Zaffar Bashir, Ali Mohd Yatoo, Fayaz Mohiddin, Neesa Majeed, Monika Bansal, Peter Poczai, Waleed Hassan Almalki, R. Z. Sayyed, Ali A. Shati, Mohammad Y. Alfaifi

**Affiliations:** 1 Centre of Research for Development, University of Kashmir, Srinagar 190006, India; 2 Department of Environmental Science, University of Kashmir, Srinagar 190006, India; 3 Mountain Research Center for Field Crops, Khudwani, Sher-e-Kashmir University of Agricultural Sciences and Technology, Srinagar 190025, India; 4 SIILAS Campus, Jaipur National University, Jaipur 302025, India; 5 Finnish Museum of Natural History, University of Helsinki, FL10044 Helsinki, Finland; 6 Department of Pharmacology, College of Pharmacy, Umm Al Qura University, Makkah 77207, Saudi Arabia; 7 Department of Microbiology, PSGVP Mandal’s, S I Patil Arts, G B Patel Science & STKV Sangh Commerce College, Shahada 425409, India; 8 Biology Department, Faculty of Science, King Khalid University, Abha 9004, Saudi Arabia

**Keywords:** biotechnological potential, cold-active enzymes, extremophiles, industrial applications, psychrophiles

## Abstract

More than 70% of our planet is covered by extremely cold environments, nourishing a broad diversity of microbial life. Temperature is the most significant parameter that plays a key role in the distribution of microorganisms on our planet. Psychrophilic microorganisms are the most prominent inhabitants of the cold ecosystems, and they possess potential cold-active enzymes with diverse uses in the research and commercial sectors. Psychrophiles are modified to nurture, replicate, and retain their active metabolic activities in low temperatures. Their enzymes possess characteristics of maximal activity at low to adequate temperatures; this feature makes them more appealing and attractive in biotechnology. The high enzymatic activity of psychrozymes at low temperatures implies an important feature for energy saving. These enzymes have proven more advantageous than their mesophilic and thermophilic counterparts. Therefore, it is very important to explore the efficiency and utility of different psychrozymes in food processing, pharmaceuticals, brewing, bioremediation, and molecular biology. In this review, we focused on the properties of cold-active enzymes and their diverse uses in different industries and research areas. This review will provide insight into the areas and characteristics to be improved in cold-active enzymes so that potential and desired enzymes can be made available for commercial purposes.

## 1. Introduction

Microorganisms are ever-present in Mother Nature and can be isolated from different environments, with features such as extreme temperatures, high salinity, water deficiency, and varying pH. Inhabiting environments with such harsh conditions, microorganisms have developed adaptive mechanisms to function under extreme circumstances. Microorganisms living and proliferating in these hostile environments are called extremophiles, and those having the ability to withstand and proliferate in multiple harsh conditions are called polyextremophiles. Microbes populating cold environs, such as bacteria, archaea, protists, unicellular algae, and fungi, are subjected to several stresses, and they have developed numerous physiological and molecular strategies to counter these circumstances [1,2,3]. These extremophiles can grow optimally in the strictest and most hostile conditions of the Earth, with the major extreme factors under which extremophiles survive and grow as follows: temperature (−2 to 20 °C, psychrophiles; 60–115 °C, thermophiles), pH (<4, acidophiles; >9, alkaliphiles), and salinity (2–5 M NaCl, halophiles) [4]. The competencies of extremophiles to thrive in these challenging environmental conditions have fascinated researchers widely. Psychrophiles were reported back in 1884, but due to the reduced focus of researchers on extreme-cold environments, and especially on pure psychrophiles, little data on psychrophiles are available from earlier times.

Microorganisms flourishing in tough cold environments have been reported from time to time; psychrophiles are habitants of environments such as the Siberian permafrost, which is considered a distinctive environment, having permanently icy grounds, limited availability of organic matter, low water activity, and additional factors [5,6,7,8,9]. Among psychrophilic microorganisms, bacteria and fungi have been isolated and investigated comprehensively as compared to yeasts, which constitute a minor part, but all forms have the potential of producing essential psychrozymes [10,11]. Cold-active enzymes retain high potential and wide implementations in food biotechnology [12]. As per recent assessments, it is forecasted that the enzyme marketplace is developing immensely and that the worldwide enzyme market may reach USD 13 to 14 billion in 2025. This is due to the specific activity of these enzymes and the high demand from different industries. Enzymes are utilized in different biotechnological and industrial areas, such as molecular biology, detergents, food and beverage processing, textile industries, and the medical and bioremediation sectors [13,14]. This review aims to cover topics relevant to prospective biotechnological uses of psychrozymes in diverse areas and to provide a better understanding of psychrophiles and their habitats.

Psychrophiles produce enzymes with about tenfold increased specific activity in this temperature range to compensate for the slow reaction rates at low temperatures [11,14]. The maximal activity for these enzymes is shifted toward the low-temperature range, showing the poor stability of these proteins and their unfolding and inactivation at mild temperatures. Such high activity at low temperatures is achieved by destabilization of the active site or maybe the whole protein, which allows the catalytic center to be more flexible at temperatures that tend to freeze molecular motions and biochemical reactions at low temperatures [14]. Psychrophilic enzymes improve the flexibility of the structural elements that are involved in the catalytic cycle, thereby resulting in an activity that is markedly heat labile.

Low temperatures are required for industrial processes that involve heat-sensitive, volatile components or in which undesirable chemical side-reactions occur at high temperatures and contamination problems must be avoided, e.g., during the manufacture of many foods, beverages, fine chemicals, and pharmaceuticals [11]. Furthermore, in addition to extremes of temperature, many industrial processes are also carried out under extremes of pH, pressure, salinity, and/or in the presence of detergents, non-aqueous solvents, among others [13].

## 2. Psychrophiles: Habitat and Potentiality

**Figure 1:**
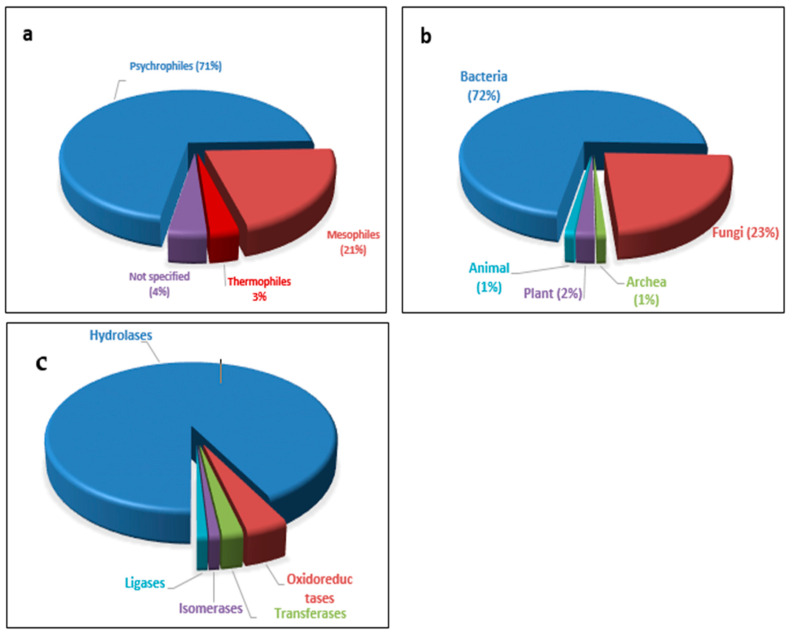
Distribution of cold-active enzymes (**a**) based on organism nature, (**b**) on organism type, (**c**) and on cold-active enzymes reported.

The diversity of microbial life inhabiting harsh or extreme environmental conditions has been extensively studied in past decades. Psychrophiles or cold-loving microorganisms belong to three domains of existence, which include archaea, bacteria, and eukaryotes; they use the extensive unpredictability of linked chemical reactions or metabolic pathways, including photosynthesis, chemoautotrophy, and heterotrophy. Different cold habitats, including both artificial and natural ones, cover about 85% of our planet, and in these habitats, a diverse range of psychrophilic microorganisms proliferates—predominantly archaea, bacteria, fungi, yeasts, and viruses—which are further categorized as psychrophiles, i.e., cold-loving microorganisms, and psychrotrophic, i.e., cold-tolerant microorganisms [9]. Psychrophilic microorganisms, especially bacteria, are considered the main source of cold-active enzymes (Figure 1a,b). Microorganisms have been differentiated based on the cold habitats in which they thrive. These environments are categorized into two groups, comprising both psychrophiles having optimum temperature for progression at about 15 °C or lower and those with maximal temperatures for progression at about 20 °C, with the minimum temperature for growth at 0 °C or lower. By contrast, the psychrotolerant ones are those microorganisms having ideal temperature for growth at about 15 °C or above, with their maximum temperature for growth being >20 °C [15,16,17]. Psychrophiles constitute about three-fourths of the biosphere and are frequently found in mountains, glacial provinces, deep-sea waters (both fresh and marine), and the Antarctic and high-altitude soils.

The majority of the biosphere of the Earth is cold, including the Arctic and Antarctica, permafrost, non-polar regions, and deep oceans [18,19]. Life has been found at temperatures as low as −32 °C and that, too, with metabolically vigorous bacteria [20]. Multiple reports are available on the isolation of psychrophiles from extreme low-temperature ecosystems. Presently the bacterium *Planococcus halocryophilus* Orl, isolated from Arctic permafrost, has been grown at the lowest temperature of −15 °C, and it is the lowest growth temperature authenticated to date [21]. For instance, the cold-loving yeast *Cystofilobasidium capitatum* SPY11 showed growth up to 20 °C isolated from and discovered in the soil of the Northern Province of India, Kashmir valley [22]. Being inhabitants of cold environments, psychrophiles have a high potential for producing industrially important enzymes, and these enzymes are moderately capable of withstanding the decrease in chemical reaction rates prompted by lower temperatures [23]. Furhan [24] reviewed current sources of important cold-adapted protease producers, their molecular adaptation, and industrial potential. The most discovered potential use of extremophiles is through their biocatalysts, organic acids, proteins, bioactive compounds, and antimicrobial agents [25,26,27,28,29]. These psychrophilic microorganisms have high potential in nutrient recycling, mineralization of organic matter, and detoxification of different metals [30,31]. With the advent of time, more focus has been given to cold-adapted microorganisms and their industrially important enzymes [32].

Psychrophiles can produce efficient and sole biocatalysts or biomolecules such as cold-active enzymes and antimicrobial compounds, and now they are receiving increasing attention from the scientific world [17,33,34,35,36]. Cold-active enzymes (CAEs) such as pectinases, proteases, amylases, lipases, cellulases, cold-shock proteins (CSPs), cold-accumulation proteins (CAPs), and ice-binding proteins (IBPs) from psychrophilic microorganisms establish a noticeable resource for biotechnological applications [37]. All components of the cell are organized correctly so that they can work at low temperatures, and the antifreeze proteins assist in avoiding ice crystal formation [38]. Long-standing adaptations of microorganisms result in genomic amendments that lead to the production of enzymes that are active at low temperatures [39]. A wide range of enzymes (pectinases, amylases, lipases, and proteases) are vastly used at the commercial level, and they have been sourced from cold-adapted microorganisms [40]. However, hydrolases are the preferred cold enzyme class discovered so far (Figure 1c). As per reports, the global market of industrially important enzymes expanded to more than USD 5.5 billion in 2018 [41]. The international marketplace for foodstuff enzymes alone expanded to USD 1.8 billion in 2017 [42]. Therefore, it is apparent that enzymes are highly used in different industrial processes and are accruing demand over time.

Enzymes from psychrophilic microorganisms are applied as additives in cleaning products; as flavoring agents in dairy and bakery products, and utilized in bioremediation processes, molecular biology, pharmaceutical, medical sciences, and biotransformation processes [2,43,44,45,46,47]. Such significant properties of psychrozymes have fascinated investigators, drawing them to work on psychrophiles and their promising metabolites.

## 3. Biotechnological Importance of Cold-Active Enzymes

Over the last few decades, the scientific and industrial communities have been exerting high efforts to discover novel cold-active enzymes that possess potential properties that can be used in multiple biotechnological processes. Microbial enzymes hold diversity and uniqueness in their properties, e.g., stability, high productivity, eco-friendliness, economically realistic nature, and high flexibility, among others; such properties in microbial enzymes are becoming a reason for their gaining importance within different industries [11,48]. The vital characteristic of psychrozymes is elevated activity at lower temperatures and thermolability; these features of cold-adapted enzymes can be utilized in molecular biology, detergents, and food and drink preparations [49,50,51]. Furthermore, the use of thermo-sensible enzymes permits their selective inactivation in complex mixtures [52]. These properties make them very attractive and sought after in multiple biotechnological uses, where they can carry out processes more efficiently, with notable cost-effectiveness, and eco-friendliness compared to high-temperature-adapted enzymes [53]. The prospective use of cold-loving microbes and their enzymes has been reviewed by different researchers [36,54,55,56].

**Figure 2:**
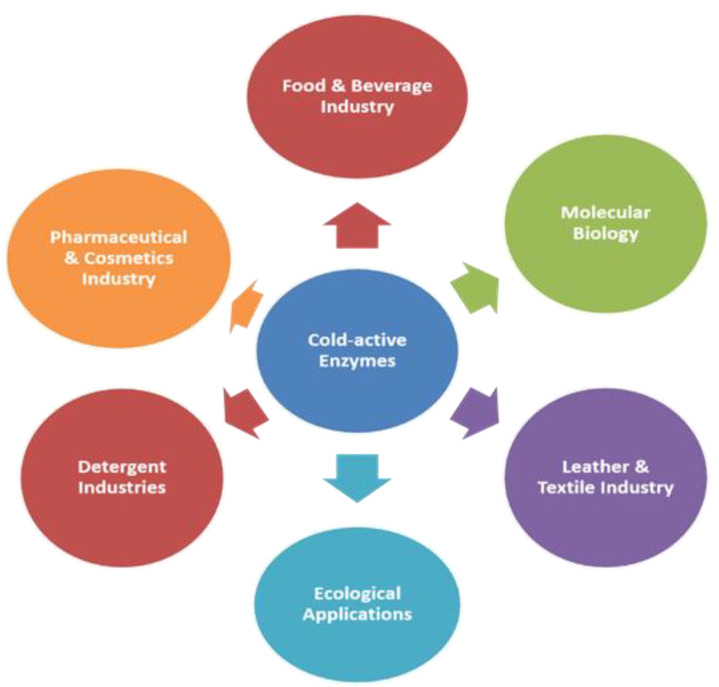
Application areas of cold-active enzymes (CAEs).

Psychrophiles (including bacteria, fungi, and yeasts) that produce cold-active enzymes have found service in numerous areas (Figure 2). Psychrozymes e.g., amylases, cellulases, invertases, proteases, and lipases, are applied in different processes carried out in the food, beverage, biofuel, and detergent industries [12,33]. Cold-active β-galactosidases have been utilized in food, cosmetic, and pharmaceutical products as well as in the production of lactose-free dairy products [11]. These enzymes are characterized based on their maximum catalytic activity at lower and adequate temperatures, though they are also heated labile and get inactivated expeditiously at mild temperatures. Cold-active enzymes have shown significant biocatalytic activity at low and moderate temperatures as compared to their mesophilic and thermophilic counterparts [30,57]. The utilization of psychrozymes in lower-temperature processes preserves heat-labile compounds. The most recent omics-era trend involves enzyme bioprospecting research using ecological metagenomics [58] and functional genomics [59]. Metagenome mining, particularly when used on microbial communities in harsh conditions, has the potential to uncover new types of enzymes while avoiding the procedural difficulties of cultivating extremophiles. However, the successful discovery of potential enzymes from environmental metagenomes continues to be difficult. As a result, functional genome mining is a viable option. The number of bacterial genomes made publicly available has exploded thanks to breakthroughs in high-throughput sequencing techniques, now numbering in the tens of thousands [60]. However, to perfectly accommodate industrial uses, cold-adapted enzymes must enhance their activity, specificity, and stability. The molecular mechanisms responsible for cold-adapted enzymes’ thermostability and activity have proven significant for the implementation in protein engineering technologies that will improve some of their features.

Residue substitutions reducing the approachability of the active site in hyperthermophilic homologs are utilized to enhance the temperature constancy and activity of cold-tolerant citrate synthases [61]. For example, single-point mutation I137M in the mesophilic *Bacillus subtilis* lipase LipJ resulted in a 17 °C drop in the ideal activity and cold adaptation [62]. The massive aromatic remains or residues on the substrate-binding sites of a psychrophilic alkaline phosphatase were substituted with more flexible amino acids in a triple mutant, resulting in an enzyme with enhanced stability while retaining the psychrophilic nature of the wild-type enzyme. Site-directed mutagenesis of cold-adapted endo-1,5-L-arabinanase has also enabled the activity’s optimal pH to be shifted near acidic settings, allowing it to be used in the extraction of pectin and clarification of juice [63]. The use of directed evolution has been the most efficacious technique for designing innovative cold-tolerant enzymes and optimizing the properties of enzymes derived from species that live in cold settings. The results of this technique usually show that cold adaptation of enzymes can be accomplished in a variety of ways. Mutagenesis and low-temperature activity assays were persuaded chemically by the mesophilic alkaline serine protease subtilisin, which results in the generation of two different triple mutants with substitution in different protein regions, each of which improved catalytic activity due to mutation.

## 4. Scope of Cold-Active Enzymes in Industries

### 4.1. Food and Brewing Industry

The application of mesophilic enzymes in the food and brewing industries has been in practice for a long time; however, the substitutions of these enzymes by their psychrophilic counterparts are providing promising results. The application of psychrozymes in food industries is proving to be a valuable asset as they are highly energy-efficient and thus help in the conservation of energy. Lactases are used in the dairy industry; as per recent investigations, a comparative study of numerous marketable β-galactosidases showed that these enzymes are effectively operational in milk at lower temperatures that lead to lactose breakdown [45,64]. The utilization of psychrophilic β-galactosidases might streamline and ease the process and budget of commercially developed lactose-free products. An Antarctic marine bacterium, *Pseudoalteromonas haloplanktis,* has been reported to produce a cold-active β-galactosidase with high efficacy in the hydrolysis of lactose under refrigerated conditions; this property will address the issue of lactose intolerance [65]. Hamid et al. [66] reported two cold-loving yeast isolates producing psychrophilic lactases capable of hydrolyzing lactose at low temperatures.

The use of cold-active β-galactosidase (temperature optima 15–18 °C) produced by psychrophilic microorganisms has opened innovative areas of research in the dairy and food processing sector with diverse biotechnological applications at low-temperature processing. Enzymes are utilized in dairy industries for cheese manufacturing and other preparations of dairy products. For instance, lactase from the yeast *Kluyveromyces lactis* is used for the hydrolysis of milk sugar (lactose) for the production of lactose-free dairy products [67]. Due to multiple properties, β-galactosidases retain abundant potential in industrial and biotechnological sectors [45,46]. Low-temperature processing in food and brewage industries is highly favorable as it provides many fruitful advantages, e.g., the prevention of bacterial contamination and spoilage, preventing the occurrence of unwanted chemical responses that can come into the picture at advanced temperatures, superior food quality, and persistence of flavor as heat-labile flavor compounds are not degraded [68]. In addition to these benefits, a major advantage of using cold-adaptive enzymes in the food industries is the ease with which they can be separated from a reaction process by simply supplying elevated temperatures [69]. Psychrozymes thus find different uses in the food and brewing industries, as do proteases in meat tenderization, pectinases in the fruit juice industry, amylases and xylanases in the baking industries, and lactases in the dairy industry [11,30].

The use of cold-active enzymes holds great industrial potential in terms of energy savings. The kinetic parameters of cold-active enzymes have shown that their kcat at low temperatures is similar to those observed for mesophilic enzymes. They function by lowering the reaction temperature without sacrificing their activity. These enzymes also prevent undesirable chemical reactions from occurring at elevated temperatures. The Km value of cold-active hydrolases from different bacterial sources has been reported between 0.3–1.01 mg/mL kcat with various substrates. While its Vmax has been reported to be between 236–97,951 mmol/min/mg.

Many psychrophilic microorganisms produce lipases; they are abundantly used in the food industry as well as in other industrial sectors. A recombinant cold-active lipase was isolated by transferring the lipase gene from *Aeromicrobium* sp. into *E. coli*; the gene product presented high stability and catalytic activity at low temperatures [70]. Cold-active proteases extracted from *Chryseobacterium* sp. can be used in meat tenderization at lower temperatures [71]. Cold-active polygalacturonase isolated from *Pseudoalteromonas haloplanktis* can be used to degrade pectin in juice manufacturing industries [72]. A report suggests that cold-active pectinases obtained from fungi and yeasts may help promote the aromatic aspects of wines by increasing the production of volatile compounds [73]. The cold-active pectinase reduces the viscosity in juice products, making preservation for a longer period possible without any mesophilic contamination [55,74]. Psychrozyme α-amylase has been reported from *Microbacterium foliorum* GA2, which was isolated from the Gangotri glacier [75], and similarly, cold-active α-amylase from marine bacterium *Zunongwangia profunda* [76] has been reported; both of them demonstrate vital features that can be used in the food and beverage industry. Other than this, ice-binding proteins from psychrophiles decrease tissue damage caused by ice crystals so that they can be significantly used in frozen foodstuffs and for cryopreservation in the biomedical sector [46]. Many other cold-active enzymes such as esterases, chitinases, and β–galactosidase have important applications in the food and brewing industries (Table 1).

Alkaline phosphatase isolated from Vibrio has an optimal temperature of 20–40 °C, and at 37–50 °C, it loses about 80% of activity at 60 °C [115], while alkaline phosphatase from the hyperthermophilic bacterium *T. maritima* has a temperature optima at 65 °C [116]. However, both are not cold-active, choose a hyperthermophilic bacterial source, and state the kinetics and temperature optima in comparison with known forms.

### 4.2. Detergent and Cleaning Industry

A sustainable approach is needed to replace harmful chemicals with enzymes that can be used in detergent industries [117]. Proteases retrieved from microorganisms are used in different industries as they account for more than 60% of overall enzyme sales in the worldwide enzyme market. Cold-active enzymes are used as detergent flavorings and possess much scope in the commercial sector as they are eco-friendly and sustainable [118]. Proteases are found to be significant enzymes in detergents and washing powders as they possess a major share in the enzyme market [117,119]. Many cold-active proteases have shown unparalleled stability and activity in a wide-ranging alkaline pH, in addition to their compatibility with detergents [102,103,120]. Proteases isolated from *Acinetobacter* sp., *Bacillus* sp., *Planococcus* sp., *Pseudomonas aeruginosa*, and *Serratia marcescens* can be used as detergent additives for cold washing [103,120,121,122,123]. Mesophilic enzymes have found utility in several industries ranging from the detergent industry and food industry to the brewing industry, and many others, owing to their superior cleaning efficiency and ecological benefits in comparison to chemical agents. From long back, proteases have been comprehensively utilized in the laundry, cleansing agent, and food processing sectors [124]. Cold-adaptive enzymes are the next level of technological programs as they not only provide these benefits but are also energy efficient. The use of cold-adaptive enzymes in cleaning processes ensures reduced wash temperatures and thus leads to the conservation of energy to a good extent. A report confirms that a mere 10 °C reduction in wash temperature produced a 30% reduction in the consumption of electricity [125].

Different cold-active enzymes, such as proteases, amylases, and lipases, have proven to be highly efficient in cleaning processes at low temperatures. Solid objects that cannot be heated for washing purposes can be easily cleaned by wipes or different formulations containing psychrozymes. The utilization of lipases in detergents or cleansing agents will increase the efficiency of the detergents in removing stains [88]. In the case of food industries, cold-active enzymes are ideal for cleaning and washing purposes as they can cut the energy stipend and downtime to a great extent [126]. The psychrozymes thus also have boundless potential in cleaning and washing processes; however, genetic alterations must be made to increase their shelf life. For instance, subtilisins obtained from Antarctic *Bacillus* sp. were engineered to increase their shelf life and were used in cold-active detergents [127]. Implementation of cold-active enzymes in detergents has improved the quality of products.

### 4.3. Pharmaceutical, Medicine, and Cosmetics

Various components used in medicine, cosmetics, fragrances, and pharmaceutical products are highly thermo-labile; the psychrophilic enzymes have structural flexibility and can operate at low temperatures, thus thermo-labile ingredients can be better synthesized by using cold-active enzymes [128]. These enzymes are used in the semi-synthesis or enzymatic modification of fine chemicals and drugs [129]. Cold-adapted enzymes have strong efficacy in pharmaceuticals, e.g., cold-adapted dehalogenases, have abundant implications for the synthesis of optically pure drug intermediates such as halo-alkanoic acids [130]. In addition, cold-adapted lipases obtained from *Candida antarctica* retain different applications, including increasing the quality of beauty products, synthesis of optically active drug intermediates, modifying sugars and their related compounds, and resolution of racemates, such as amines and alcohols, during the formation of various cosmetic products (i.e., iso-propylmyristate), fragrance esters, and pharmaceuticals (i.e., NK1/NK2 antagonist used for asthma treatment) [131,132]. The psychrophilic enzyme β-galactosidase, is used for the synthesis of galactooligosaccharides, and tagatose is used as prebiotic and antihyperglycemic agents, respectively [133].

Moreover, cold-tolerant protease is used for the synthesis of bioactive peptides used as an antioxidant and antihypertensive agent. Due to the antimicrobial activity of cold-adapted protease, it is used against viral and bacterial infections as a therapeutic agent and is also used in cosmetics for the removal of dried and dead skin cells [134,135]. The other example of cold-adapted enzymes used in biomedicine includes nitro-reductase and α-galactosidase. The former is used as cancer pro-drug activating enzyme, whereas the latter can be used in blood transfusion therapy [136,137]. It has been shown that psychrozymes isolated from psychrophiles have great medicinal importance.

### 4.4. Molecular Biology

Cold-loving microorganisms and their enzymes hold colossal functions in the area of molecular biology. Researchers working on cold-active enzymes are modifying and improving features of isolated wild strains of cold-loving microorganisms to get a desired cold-active enzyme with multiple characteristics [2]. This progression in enzyme technology is only possible with the intervention of different tools of genomics. Another prominent cold-adapted enzyme used at the molecular level is alkaline phosphatase; it is employed for the removal of phosphate groups from the 5′ ends of DNA molecules and, thus, prevents self-ligation of DNA molecules. For commercial purposes, the enzyme is obtained from *E. coli* and calf duodenal tissue. The alkaline phosphate obtained from *E. coli* and calf intestinal tissue is heat stable, and hence extraction is performed via inorganic methods [138]. Cold-adapted alkaline phosphatases, both single- and double-stranded nucleases, and uracil-DNA N-glycosylases are recently being commercialized as a molecular biological tool by various companies (New England Biolabs Inc., ArcticZymes, Takara-Clontech, Affymetrix, Inc.) [139,140,141]. The Antarctic marine bacterium, *Vibrio* sp., was isolated and used for the production of heat-labile alkaline phosphatase. This enzyme has a higher turnover number (k(cat)) and higher apparent Michaelis–Menten factor (K(m)) as compared with enzyme from *E. coli*, a clear indication of cold-adaptation [108]. An Arctic shrimp heat-labile alkaline phosphatase was used for commercial purposes [142]. Two more heat-labile psychrophilic enzymes are available for their utilization in molecular biology. They are shrimp nuclease and heat-labile uracil-DNA N-glycosylase, both capable of contaminant removal and DNA helix degradation during PCR. The former is used as a recombinant form in *Pichia pastoris*, and the latter is obtained from Atlantic cod (*Gadus morhua*) and used as a recombinant form in *E. coli.* Both psychrozymes can be easily inactivated by moderate thermal treatment [127].

To achieve sufficient enzyme yield with efficient enzymatic properties, it is important to develop recombinant countenance in a heterologous host. Mesophilic hosts are frequently employed for recombinant expressions at the genomic level to encode psychrozymes, although these microbes’ ideal growing temperature is incompatible with the temperature at which cold-active enzymes require correct folding to maintain their arrangement and function [143]. Several novel strategies are adopted nowadays for improving the expression to improve their solubility, protein yield, and proper folding of cold-active enzymes.

The isolation of a cold-adapted bacterium with a unique enzymatic activity served as the starting point for enzyme cloning. Designing customized primers for gene intensification using the strain’s DNA as a template is the most common cloning approach. However, this approach is feasible only when the genomic sequence has already been deposited in Gene Bank and if the microbe can be easily cultured to isolate its genetic material. Another option is to create a gene with the finest codon norm for the host. The selected expression host in most cases was BL21 (DE3) as the preferred strain. Other expression hosts were *Halobacterium* sp., which expresses a cold-adapted hydrolase, and *Pichia pastoris*, which is employed as an expression host for a variety of cold-adapted proteins. For expression, the majority of these genes were cloned on plasmids from the pET system. Fusion constructs were also used for cloning many cold-adapted genes; pGEX-6P-1 allows proteins to be expressed in fusion with GST and pGEX-6P-2 in pMAL-c, which expresses protein fusion to MBP.

The chaperones Cpn60 and Cpn10 from the cold-loving bacterium *Oleispira antarctica* RB8 were expressed using *E. coli* as reported by Ferrer and colleagues in 2004 [144]. They successfully expressed a heat-labile esterase using this chaperone *E. coli* system, establishing a viable expression strategy for heat-sensitive proteins. They also discovered that, when compared to the enzyme extracted from a standard *E. coli* strain cultivated at 37 °C, lower temperature improves enzyme folding and increases specific activity 180-fold.

Agilent Technologies now sells Arctic Express, which is a competent *E. coli* strain that co-expresses the cold-active chaperones Cpn60 and Cpn10. In an *E. coli* strain, Kim et al. [145] found that PsyGroELS, a chaperonin from the psychrophilic bacterium *Psychrobacter* sp. PAMC21119, co-expressed a cold-active esterase. In comparison of enzyme activity with that of previously described chaperones Cpn60 and Cpn10, PsyGroELS produced better results. Using GroES/GroEL chaperones for protein production, Esteban-Torres et al. [146] copied the cold-active esterase lp 2631 into the pURI3-TEV expression vector, but the recombinant protein was expressed as inclusion bodies in *E. coli* BL21 (DE3). They employed the plasmid pGro7, which produces GroES/GroEL chaperones, to fix the problem. Different tools of molecular biology and recombinant DNA technology can be used to study the mechanism of cold-adaptation among psychrophiles, and this may lead to the formulation of novel cold-active enzymes with fascinating properties.

### 4.5. Bioremediation or Ecological Applications

Microbes have been astounding environmental cleaners from the dawn of civilization and have provided a major hand in remediating the environment. Using efficient psychrozymes in bioremediation own several advantages, as in cold climatic environments it is difficult for the whole cell to face additional challenges, so utilization of cold-active enzymes may help in meeting clean-up standards in a short time [99]. A report published in 2014 demonstrated the presence of hydroxylase genes in psychrophilic microorganisms; these genes help them to degrade crude oil and alkane into ecologically neutral compounds [147]. These psychrophiles will provide a massive advantage as they can be used easily to check the petroleum pollution in colder regions. Because of low temperatures in cold environments, it is challenging for microorganisms to meet clean-up standards for bioremediation as it consumes more time [99]. As whole bacterial cells need multiple parameters for optimal growth and metabolic activities that are lacking in groundwater, the use of enzymes could be more feasible and result-oriented than the use of whole bacterial cells [148]. In another report, psychrophilic yeasts were found to degrade phenol and other related compounds at 10 °C [149]. Many cold-adapted microbes such as *Pseudomonas* sp., *Rhodococcus* sp., and *Pseudoalteromonas* are capable of degrading petroleum hydrocarbons [150,151,152]. Psychrophiles, along with their bioremediation capacity, will help us in retaining soil health and crop productivity.

## 5. Conclusions and Future Perspectives

Microorganisms are universal, and they thrive in different environmental conditions. Microorganisms, including bacteria, fungi, and yeast, inhabiting the cold environments of the Earth are attracting the attention of microbiologists and researchers from all over the world because of their uniqueness in their metabolic activities. They have gained attention from different industries due to their cold-active enzymes. The scope of biotechnological applications of novel extremozymes from psychrophiles is expanding with time. This is because of their ability to catalyze reactions at low temperatures close to the freezing point of water and act as an energy-saving tools. The utilization of cold-active enzymes in low-temperature catalysis will help in retaining the flavor of the product and protect the process from contamination. Psychrophilic enzymes possess a vital role in industries for large-scale production, but despite great efforts, the commercialization of efficient and novel cold-active enzymes is still in its infancy. It is important to explore these potent enzymes that will be eco-friendly, economical, and efficient.

## Figures and Tables

**Table 1:** List of some cold-active enzymes and their potential industrial applications.

Cold-Active Enzyme	Source of Isolation	Potential Applications	References
Lactases/β-galactosidase	*Paracoccus* sp., *Cystofilobasidium capitatum* SPY11, *Rhodotorula* sp.	Dairy industry (lactose hydrolysis)	[11,45,66,77]
α-Amylases	*Geomycespannorum*, *Bacillus subtilis* N8, *Geomyces pannorum*	Food, baking, and detergent industries	[78,79,80]
Cellulases	*Pyrococcus* sp.	Food and textile industry, ethanol fermentation	[81,82]
Chitinases	*Metschnikowia* sp., *Glaciozyma antarctica*, *Mrakia psychrophila*, *Sporobolomyces salmonicolor*	Meat tenderization, degradation of chitin rich wastes, control of phytopathogens	[83,84,85]
Lipases	*Pseudoalteromonas haloplanktis* TAC125, *Penicillium canesense*, *Pseudomonas* sp. VITCLP4	Animal feed, detergent, and textile industries	[86,87,88]
Glycogen branching enzyme	*Rhizomucor miehei*	Wheat bread manufacturing and baking industry	[89]
Phytases	*Erwinia carotovora*, *Candida carpophila*, *Cryptococcus laurentii*, *Yarrowia lipolytica*	Food and feed industry	[90,91,92]
Pectinases (polygalacturonase and pectin-methylesterase)	*Cystofilobasidium capitatum* PPY-1, *Rhodotorula mucilaginosa* PT1, *Cystofilobasidium capitatum* SPY11, *Leucosporidium drummii*, *Sporobolomyces salmonicolor*, *Penicillium chrysogenum* F46	Food and fruit industries, pectin degradation, juice extraction	[35,93,94,95]
Xylanases	*Pseudoalteromonas haloplanktis* TAH3A, *Flavobacterium* sp. MSY-2, *Rhodococcus* sp., *Pseudomonas* sp., *Flammeovirga pacifica* WPAGA1, *Cryptococcus adeliensis*	Baking industry, xylan hydrolysis, osmoprotectants, biofuel production	[96,97,98,99]
Metalloproteases	*Pedobacter cryoconitis*	Bioremediation of wastewater at a lower temperature	[100]
Alkaline protease	*Stenotrophomonas* sp., *Bacillus subtilis* WLCP1	Detergent and textile industries	[101,102,103]
Proteases	*Flavobacterium limicola*, *Acinetobacter* sp., *Geomyces pannorum*, *Naganishia albida*	Textile and leather industries, organic polymer mineralization in freshwater sediments, food and feed industry	[104,105,106,107]
Serine proteases	*Pseudoalteromonas* sp.	Low-temperature food processing, leather industry	[63]
Alkaline phosphates	*Vibrio* sp.	Molecular biology	[108]
DNA ligase	*Pseudoalteromonas haloplanktis*	Molecular biology and recombinant DNA technology	[109]
RNA polymerase	*Pseudomonas syringae*	Molecular biology	[110]
Fuculose aldolase	*Glaciozyma antarctica* PI12	Pharmaceutical industry	[111,112]
Ectoine synthase	*Sphingopyxis alaskensis*	Cosmetics, biomedical industry	[113,114]

## Data Availability

The data presented in this study are available in this manuscript.
